# Investigation and evaluation of the efficiency of palm kernel oil extract for corrosion inhibition of brass artifacts

**DOI:** 10.1038/s41598-025-88370-0

**Published:** 2025-02-06

**Authors:** Mohamed M. Megahed, Eman AbdElRhiem, Wesam Atta, Nabil Abdel Ghany, Mohamed Abdelbar

**Affiliations:** 1 Conservation Department, Faculty of Archaeology, Fayoum University, Faiyum, Egypt; 2 Mining and Metallurgy Engineering Department, Tabbin Institute for Metallurgical Studies (TIMS), Tabbin, Helwan, 109, Cairo, 11421 Egypt; 3 Physical Chemistry Department, National Research Centre, Dokki, Cairo, Egypt; 4 Conservation Department, Faculty of Archaeology, Damietta University, Damietta, 34511 Egypt

**Keywords:** Brass alloy, Corrosion, Palm kernel oil, Electrochemical measurements, Environmental sciences, Chemistry

## Abstract

Brass (Cu–Zn alloy) used in the crescent of the Al-Maradani Mosque pulpit is subjected to corrosion under certain conditions, such as exposure to polluted air, oxidizing acids, and compounds containing sulphur or ammonia. This study aimed to evaluate the effectiveness of palm kernel oil extract (PKO) as a green corrosion inhibitor for protecting brass artifacts. The crescent was analyzed using metallographic microscopy, scanning electron microscopy (SEM) with energy-dispersive X-ray (EDX), and X-ray diffraction (XRD) to identify its alloy composition, microstructure and corrosion products. The analyses confirmed the crescent was made of a brass alloy (Cu–Zn) and formed by hammering, with corrosion layers composed primarily of cuprite and clinoatacamite, covered by dust containing calcite and quartz. The corrosion protection efficiency of the PKO was evaluated using brass coupons, simulating the artifact of the alloy. Electrochemical methods, including the open circuit potential (OCP), Tafel, and electrochemical impedance spectroscopy (EIS), were used to assess the performance of palm kernel oil on brass coupons at different concentrations (1, 3, 5, 7%). Electrochemical tests showed that corrosion inhibition efficiency increased with higher palm kernel oil concentrations, with the 7% concentration exhibiting the highest corrosion protection, up to 99.7%.

## Introduction

Corrosion poses a significant threat to the preservation of metallic artifacts, especially those in uncontrolled environments where it is difficult to eliminate. As a result, corrosion prevention is a priority in the conservation of cultural and archaeological heritage. Among the various methods used to combat corrosion, the application of inhibitors has proven to be one of the most effective strategies^1–5^.

In recent years, the focus has shifted towards the use of green corrosion inhibitors due to their eco-friendly nature and cost-effectiveness. Green corrosion inhibitors, particularly those derived from natural plant extracts, have shown great promise in protecting metals by forming a protective barrier that blocks active sites on the metal surface, thereby mitigating interaction with corrosive elements^6–12^. The advantages of using plant extracts as corrosion inhibitors are manifold: they are biodegradable, non-toxic, inexpensive, and readily available. Additionally, they do not pose risks to human health or the environment, unlike many synthetic inhibitors^13–15^. There is a wide range of phytochemicals, such as tannins, flavonoids, and polyphenols, so it has a high potential for corrosion inhibition. The first use of a plant extract as a corrosion inhibitor dates back to 1930 when it was the extract of Chelidonium majus and some other plants in a pickling bath of H_2_SO_4_^16,17^.

Numerous studies have demonstrated the effectiveness of different natural plant extracts and oils as corrosion inhibitors for the protection of copper and its alloys. Examples include Watermelon Seed Extract as a green corrosion inhibitor, which highlighted the effectiveness of watermelon seed extract in saline environments^18^. Jatropha Extract for bronze alloy corrosion, which exhibited up to 90% efficiency through adsorption mechanisms in neutral media^19^. Avocado Seed Extract, achieving an inhibition efficiency of 87% for aluminum alloys in saline solutions through the formation of a protective passive layer^20^. Mint Leaf Extract and NiO Nanoparticles, demonstrating high inhibition efficiencies on copper surfaces^21^. Aqueous Extract of Pointed Gourd Seeds (PGS), which achieved corrosion protection for mild steel^22^. Mint Leaf Water Extract for copper corrosion, showing effective mitigation of copper corrosion^23^. Opuntia Dillenii Seed Oil provides excellent inhibition efficiency of 99% for iron in acidic rain solutions by forming a barrier layer through chemisorption and physisorption^24^.

Plant extracts contain various biologically active components, such as terpenes, alkaloids, flavonoids, and phenolic compounds; otherwise, oils contain a mixture of fatty acids and other compounds, which are known for their corrosion-inhibiting properties. These compounds adsorb onto the metal surface, creating a protective film that acts as a physical barrier against corrosive agents. Despite the natural tendency of plant extracts to biodegrade, this issue can be mitigated by adding biocides, which prevent decomposition and extend the useful life of the inhibitors. The use of natural substances, particularly vegetable oils, as corrosion inhibitors has gained increasing attention. Vegetable oils are appealing due to their low cost, abundant availability, and environmental friendliness. Among them, palm kernel oil (PKO) stands out as a particularly effective inhibitor. Derived from the seed of the palm fruit, palm kernel oil is distinct from palm oil in its composition, being richer in saturated fatty acids and containing a high concentration of lauric acid. These properties make PKO more solid at room temperature and enhance its ability to form a stable, protective layer on metal surfaces^16^. Research into the use of palm kernel oil as a corrosion inhibitor has demonstrated its high efficacy in protecting metals and alloys. It is a potentially non-toxic, abundantly available, and environmentally friendly natural product. Studies have shown that palm kernel oil, when applied as a corrosion inhibitor, can achieve an inhibition efficiency of up to 97.20% under optimal conditions, which include an inhibitor concentration of 1.00 g/l, a 4-day exposure period, and a temperature of 40 °C^25–27^.

Electrochemical techniques have a long history in conservation-restoration treatments for metallic cultural heritage, particularly the use of electrochemical impedance spectroscopy (EIS). EIS is well-established for evaluating protective coatings and is especially suited for testing coatings on metallic works of art. It helps assess parameters such as corrosion inhibition efficiency and surface coverage by inhibitor films. This technique, which measures changes in polarization resistance (R_p_) and electrode capacitance, has been widely used since the early 1990s to quantify the protective effectiveness of coatings against metal corrosion, providing valuable insights for the preservation of cultural heritage^28–33^.

This study aimed to evaluate palm kernel oil (PKO) as a green corrosion inhibitor for protecting brass artifacts, specifically the Al-Maradani Mosque pulpit, from corrosion. Additionally, it involved the investigation and characterization of the brass artifact. The research offers valuable insights into the conservation of cultural heritage materials using environmentally friendly additives.

## Material and methods

### Case study

**Fig. 1:**
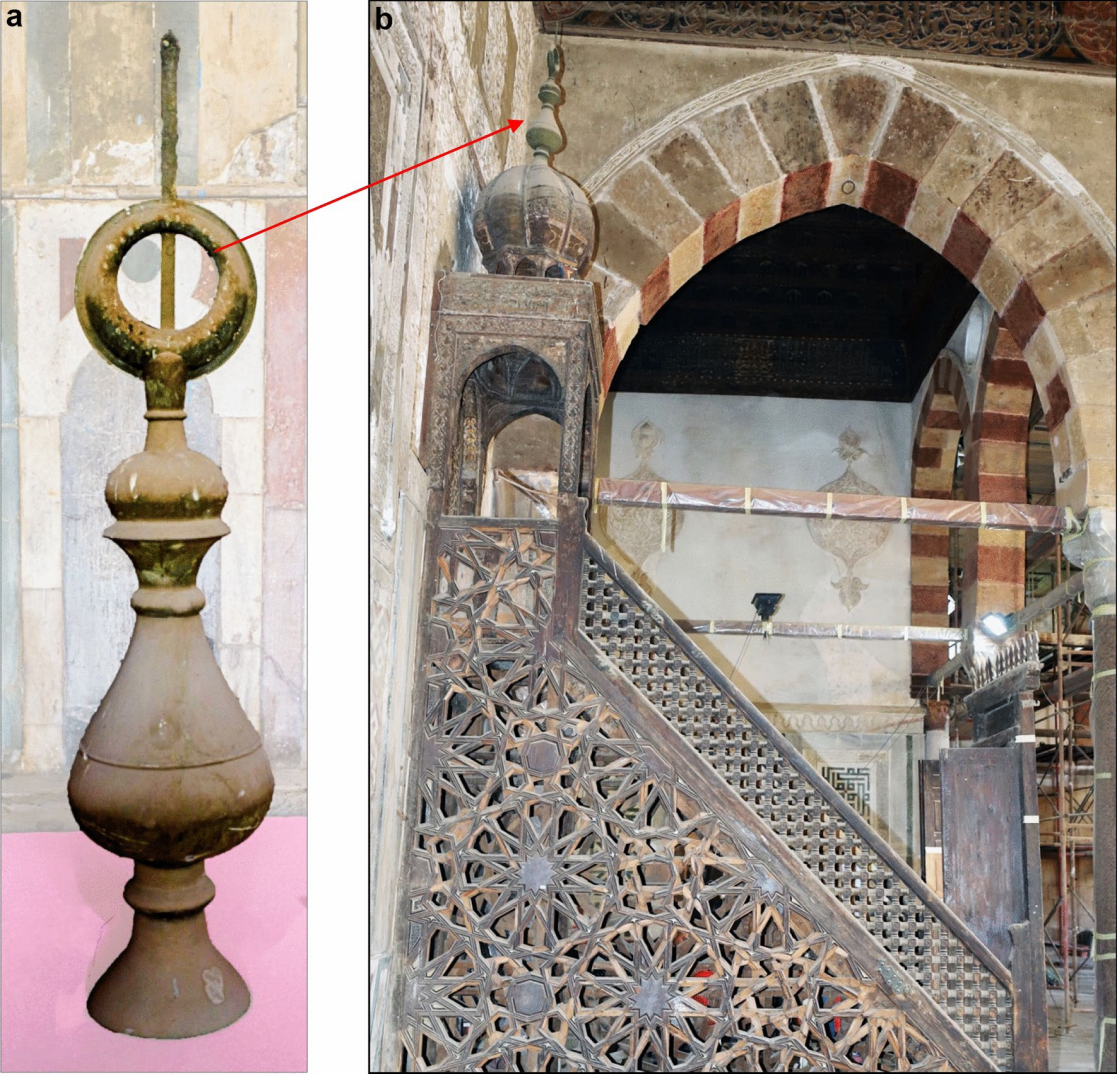
(**a**) General view of the crescent covered with thick layers of dust and bird droppings. (**b**) The crescent is situated above the pulpit pavilion.

**Fig. 2:**
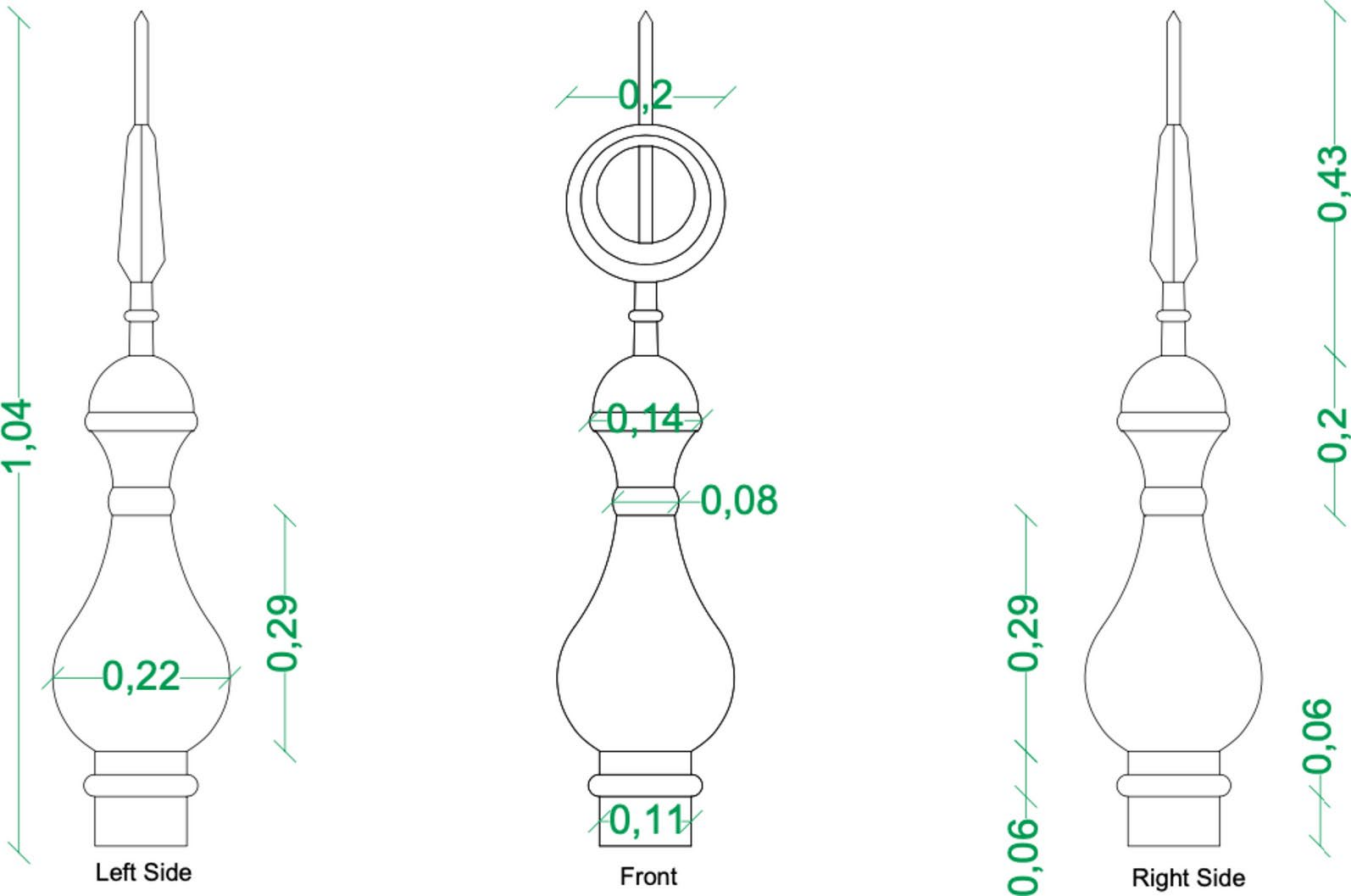
A two-dimensional drawing of the crescent was created using AutoCAD, with dimensions shown in meters.

The crescent atop the pulpit (minbar) of the Mosque of Amir Altinbugha al-Maridani, dating from 1340 CE during the Mamluk Sultanate in Cairo, Egypt, is crafted from Brass, a copper-zinc alloy. It features a circular base that rests on the dome above the minbar. From the base, a short neck leads to an elongated oval body that tapers upward. Another short neck supports two flattened circles, which are topped by a larger neck. The structure is crowned with a circle that has a wide opening, completing the design (Fig. 1a). The crescent, often placed atop domes, minarets, or the pavilion above the pulpit (minbar), is aligned parallel to the direction of the Qibla (Fig. 1b). The Ottomans were the first to introduce the crescent on minarets during the reign of Sultan Selim I, initially to differentiate mosques from churches. The crescent was positioned on the highest part of the minaret, a practice that has continued ever since. Within the mosque, the Qibla is indicated by the Mihrab, while outside, it is marked by the opening of the crescent. This is the purpose and significance of placing the crescent^34^. The Minbar is crowned by a horseshoe arch and surmounted by a dome supported by four columns, which culminate in a brass crescent (called Hilal in Arabic). The crescent weighs approximately 5780 g. Figure 2 illustrates the dimensions of the meter. It is about 104 cm in length, with a maximum width of 22 cm at its center and a diameter of approximately 11 cm at the base of the opening. The crescent’s opening has a diameter of roughly 20 cm.

The crescent was examined using various techniques to explore its alloy composition and degradation process. Surface morphology was analyzed with a Quanta FEG250 (Japan) environmental scanning electron microscope, while the alloy’s microstructure was studied using an OLYMPUS BX41M-LED metallographic microscope. Corrosion products and surface calcifications were assessed with a PANalytical X’Pert Pro X-ray diffraction system. The elemental composition of the crescent was determined using energy dispersive spectroscopy (EDS) attached to the Quanta FEG250 SEM.

### Corrosion test

#### Coupons preparation

For the simulation experiments, brass sheets (1 cm width × 5 cm length × 0.2 cm thickness) were prepared based on the chemical composition of the real brass crescent (Cu–Zn alloy). Each coupon was numbered and stamped with punches for identification. The coupons were initially polished using different grades of emery papers, rinsed with running and distilled water, then cleaned with ethyl alcohol, and thoroughly dried. Finally, they were stored in a dry environment to prevent corrosion before the experiments^4^.

#### Palm kernel oil (PKO)

The palm kernel oil (PKO) was commercially sourced from KEMATECH-ALEX, Alexandria, Egypt. Typically, PKO is prepared by first drying and then grinding the palm kernels into a fine powder. The powder is then soaked in ethanol solution for 24 h to achieve a homogenous mixture, after which the extract is collected. Any residual ethanol is removed through evaporation, leaving the filtrate to be used as an inhibitor in its purest form^27^.

##### Gas chromatography analysis

Gas Chromatography (GC) was employed to screen the biochemical composition, including major and minor components, of commercially produced palm kernel oils. The oil was first diluted to a 1:200 ratio with hexane to prepare it for injection. The analysis was carried out using an Agilent 7890B GC system, equipped with a flame ionization detector (FID), at the Central Laboratories Network, National Research Centre, Cairo, Egypt. Separation was performed on a Zebron ZB-FAME column (60 m × 0.25 mm internal diameter × 0.25 μm film thickness). The gas chromatography analysis was carried out using hydrogen as the carrier gas at a flow rate of 1.8 ml/min. The injection volume was 2 µL, operating in split mode (1:10). The temperature program began with an initial oven temperature of 100 °C, held for 3 min, followed by a 2.5 °C/min increase until reaching 240 °C, which was maintained for 10 min. The injector temperature was set to 250 °C, while the flame ionization detector (FID) was held at 285 °C.

##### Preparation of (PKO) as a corrosion inhibitor

Palm kernel oil (PKO) was diluted in a controlled manner to achieve specific concentrations for electrochemical measurements. A PKO stock solution was prepared by mixing 5 ml acetone and 5 ml ethyl alcohol, to which 10 ml of PKO was added, ensuring proper dissolution.1, 3, 5, and 7% of the stock solution was used. The working electrolyte stock consisted of a 3.5% Sodium Chloride solution.

#### Electrochemical tests

Electrochemical measurements were carried out using an Origaflex-OGF01A (Origalys, France) system. The setup included an Ag/AgCl reference electrode, a platinum sheet as the auxiliary electrode, and a brass sheet as the working electrode. Electrochemical impedance spectroscopy (EIS) was conducted at open circuit potential (OCP), while Tafel plots were used to determine the corrosion rate (C.R.) and corrosion current density (I_corr_.) at a scan rate of 2 mV/s within a potential range of -300 to 300 mV. EIS measurements were performed over a frequency range of 0.1 Hz to 100 kHz with a 10 mV amplitude, following the ASTM G106-89 standard. A 3.5% NaCl solution, prepared with triple-distilled water, served as the electrolyte for the corrosion testing. Each electrochemical measurement was conducted in triplicate to ensure accuracy and reliability.

## Results and discussion

### Brass crescent

Brass, an alloy of (copper-zinc), is highly valued for combining the beneficial properties of both metals and is widely used in various fields. Historically, techniques like smoothing and hammering brass were essential in crafting items such as crescents. Manufacturers skillfully utilized hammers, anvils, and precise procedures to form the desired shapes. Soldering played a crucial role in Crescents’ manufacturing process. Copper plates were hammered on an anvil, and the ends of the plate were joined together by solder. Brass soldering, a solid joint of copper and zinc, ensured durability. In brass artifact manufacturing, this tradition continues, where the brass sheet is flattened, hammered, and soldered using hard solder to create robust and finely shaped artifacts^35,36^.

**Fig. 3:**
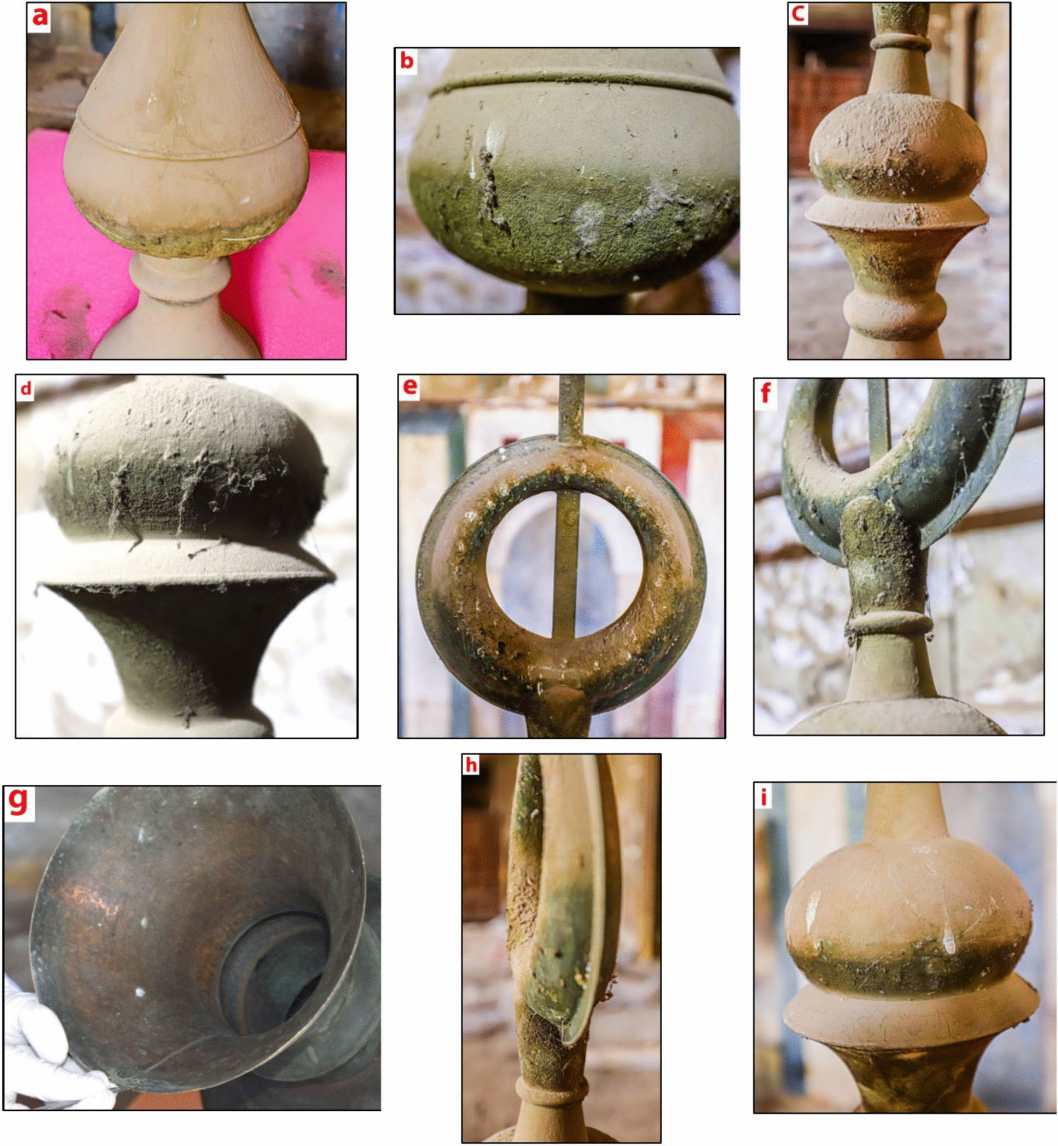
(**a**–**i**) The crescent is covered with dust, dirt, and bird excrement. (**g**) Shows the crescent’s underside.

Copper and its alloys generally exhibit high resistance to atmospheric corrosion due to the formation of protective layers of corrosion products, which slow down the rate of attack. The thickness and composition of these corrosion layers are influenced by environmental factors such as relative humidity and pollution levels. Figure 3 illustrates the extent of damage to the brass crescent. The crescent is heavily covered with dust and dirt, indicating prolonged exposure to environmental elements. The presence of these surface contaminants can accelerate corrosion processes.

#### Alloy microstructure

**Fig. 4:**
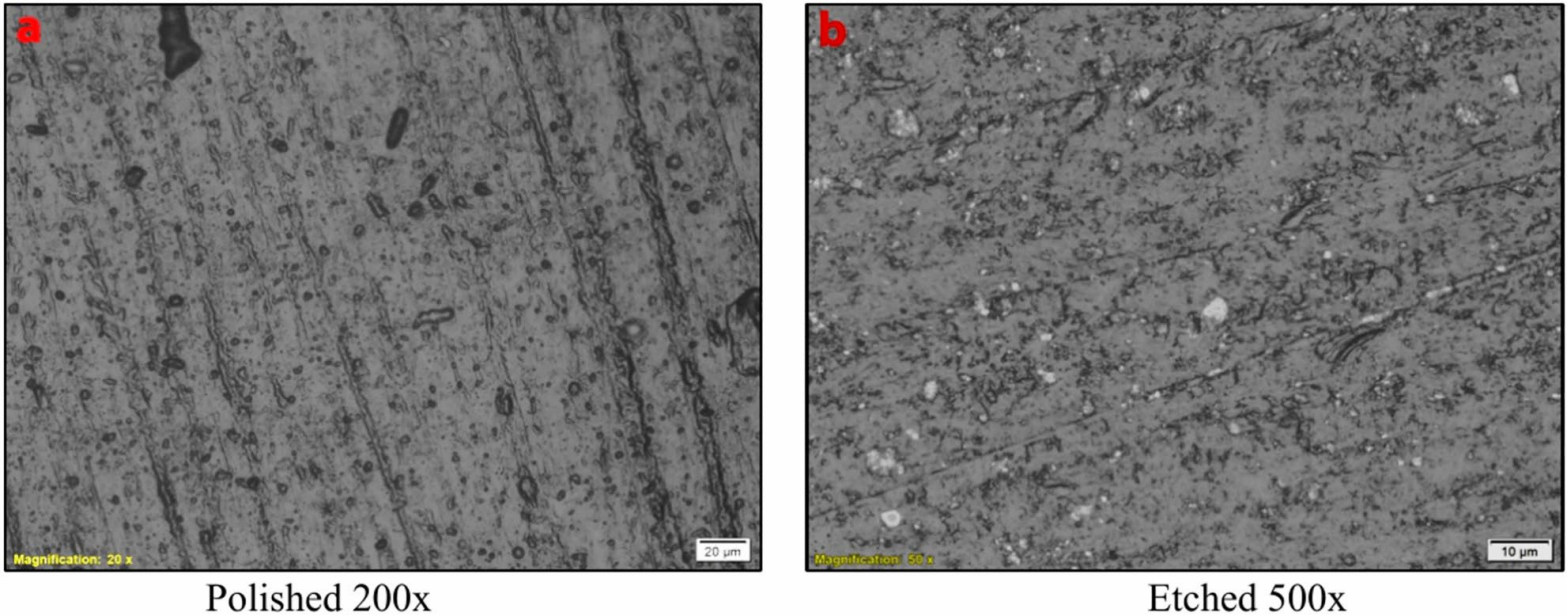
(**a**, **b**) Microstructure of the brass alloy of the crescent, showing a single-phase (α-phase) containing up to 35% zinc.

The microstructure of ancient brass alloys typically consists of a combination of α-phase (copper-rich) and β-phase (zinc-rich) regions, with varying proportions depending on the zinc content, often exhibiting grain boundaries and inclusions that reflect the alloy’s historical manufacturing techniques and usage (Fig. 4). Brass is classified by its phase: alpha brass (up to 35% zinc), α + β Brass (35–46.6% zinc), and beta brass (46.6–50.6% zinc). Alloys with more than 50% zinc are generally avoided due to the brittle δ phase, as the beta phase is harder than alpha, can endure little cold work, and becomes more malleable above 470 °C. Alpha Brass, found in most ancient alloys, is ductile, easily cold-worked, and has good corrosion resistance, which is advantageous for the longevity and preservation of artifacts. Based on the EDS analysis, the brass alloy of the crescent, containing approximately 33.65% zinc, falls within the category of alpha brass, which is characterized by a single-phase microstructure (α-phase) that is ductile and has good cold-working properties. The microstructure confirmed a homogeneous alpha brass microstructure without the presence of the (β-phase), with the alpha phase typically showing a face-centred cubic (FCC) structure that contributes to the alloy’s ductility and workability^37–41^.

#### Alloy chemical composition

**Fig. 5:**
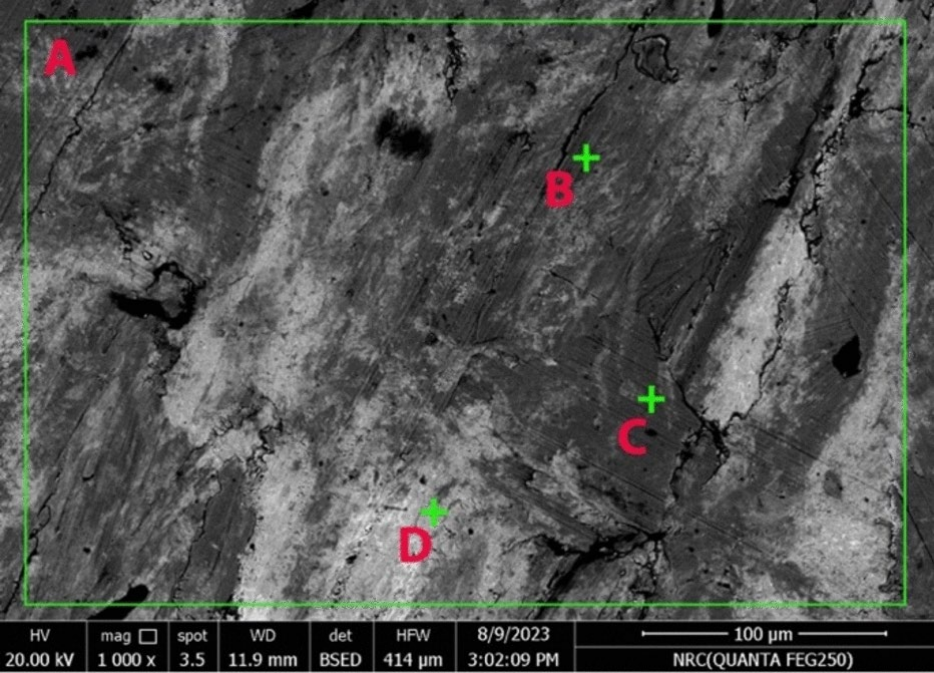
SEM image of the brass alloy of the crescent, displaying a single-phase (α-phase) containing up to 35% zinc.

SEM examination of the brass crescent revealed zinc white globules dispersed throughout the alloy, along with stress corrosion cracking (SCC), resulting in small, scattered cracks (Fig. 5). SCC is particularly common in Brass, occurring under tensile stress in polluted (industrial) environments, especially those containing ammonium compounds. This phenomenon typically affects copper alloys with 20% or more zinc but is less common in other alloys^42^.

**Fig. 6:**
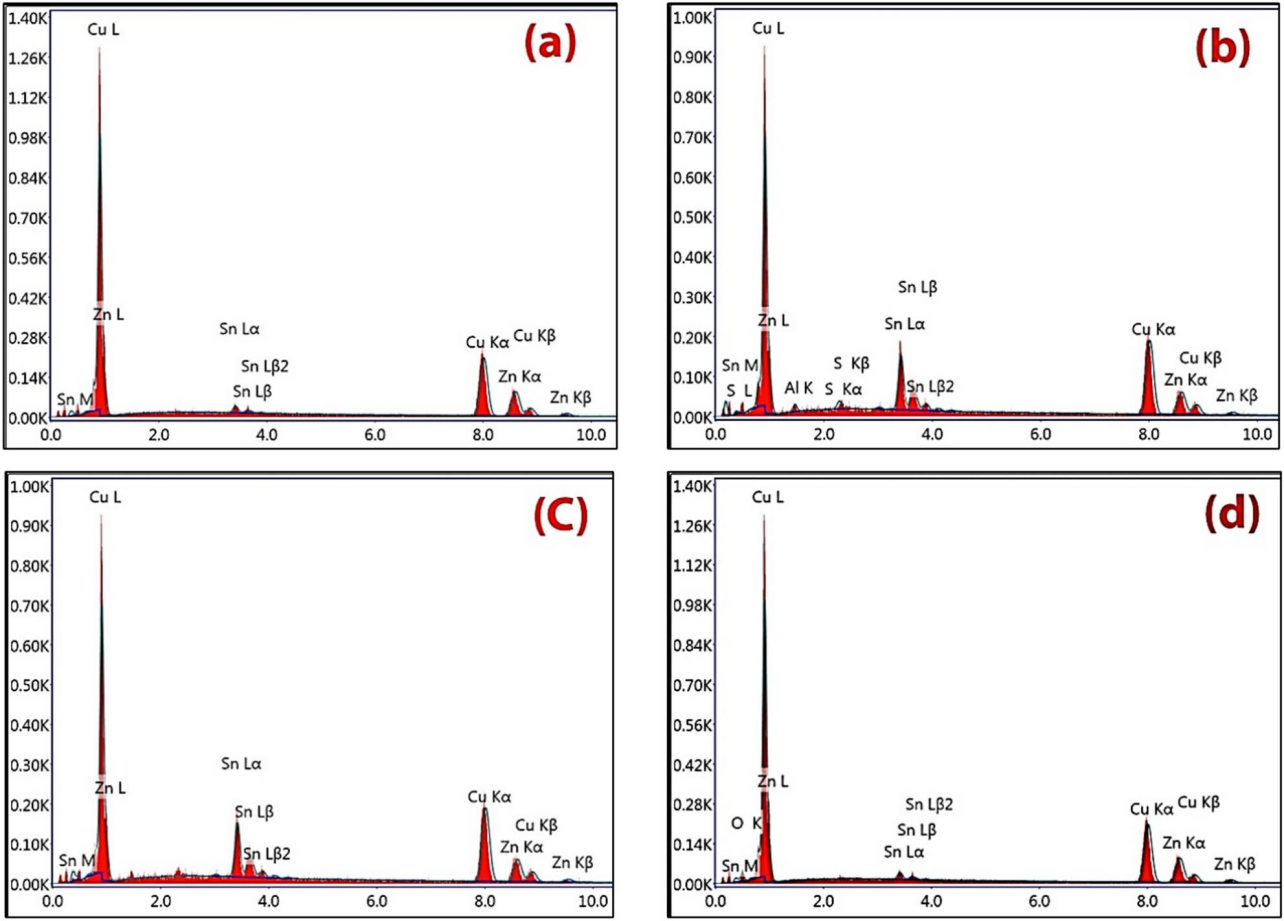
(**a**–**d**) EDS analysis of various spots (A–D) in the brass alloy of the crescent.

EDS analysis was performed to determine the chemical composition of the alloy that made up the crescent. The results revealed that the crescent was made of a brass alloy containing 64.77% Cu, 33.65% Zn, and 1.58% Sn, as shown in Fig. 6 and Table 1. Yellow brass alloys typically contain 23–41% zinc, with up to 3% lead and 1.5% tin as additional alloying elements.

**Table 1:** Results of EDS analysis of different spots in the crescent alloy.

Element	Cu	Zn	Sn	Al	S	O
A	64.77	33.65	1.58	**–**	**–**	**–**
B	63.46	19.37	11	3.81	2.37	**–**
C	67.08	21.33	11.59	**–**	**–**	**–**
D	58.72	30.47	1.46	**–**	**–**	9.35

#### Corrosion degradation

It is well known that brasses are copper-zinc alloys that are extensively used in many fields, and they combine many of the favourable features of both copper and zinc. Since zinc is the active component of brass, it tends to corrode, leaving its surface enriched with copper. The corrosion of a metal is often considered an inconvenience because it implies a change in the objects over the course of time. This damage is caused to metal by chemical, electrochemical, or even biological reactions between metal and the surrounding medium^43^. Selective corrosion occurs when one component of an alloy, such as zinc in copper-zinc alloys, dissolves preferentially, resulting in a weakened, porous, copper-rich structure. This process, known as dezincification, transforms the material into spongy copper, increasing its susceptibility to corrosion cracking. Dezincification typically affects alloys with zinc content above 15 wt.%, where copper re-precipitates after both metals dissolve^44^.

The electrical potential difference between copper and zinc, in the presence of water, oxygen, and impurities, leads to intergranular corrosion in Brass, weakening it and making it prone to stress damage. Reshaping without prior heat treatment can cause distortion, especially if microcracks are present. The corrosion rate is further influenced by the contact between metals of different electrochemical potentials, where the less noble metal becomes anodic and corrodes more rapidly^41^.

**Fig. 7:**
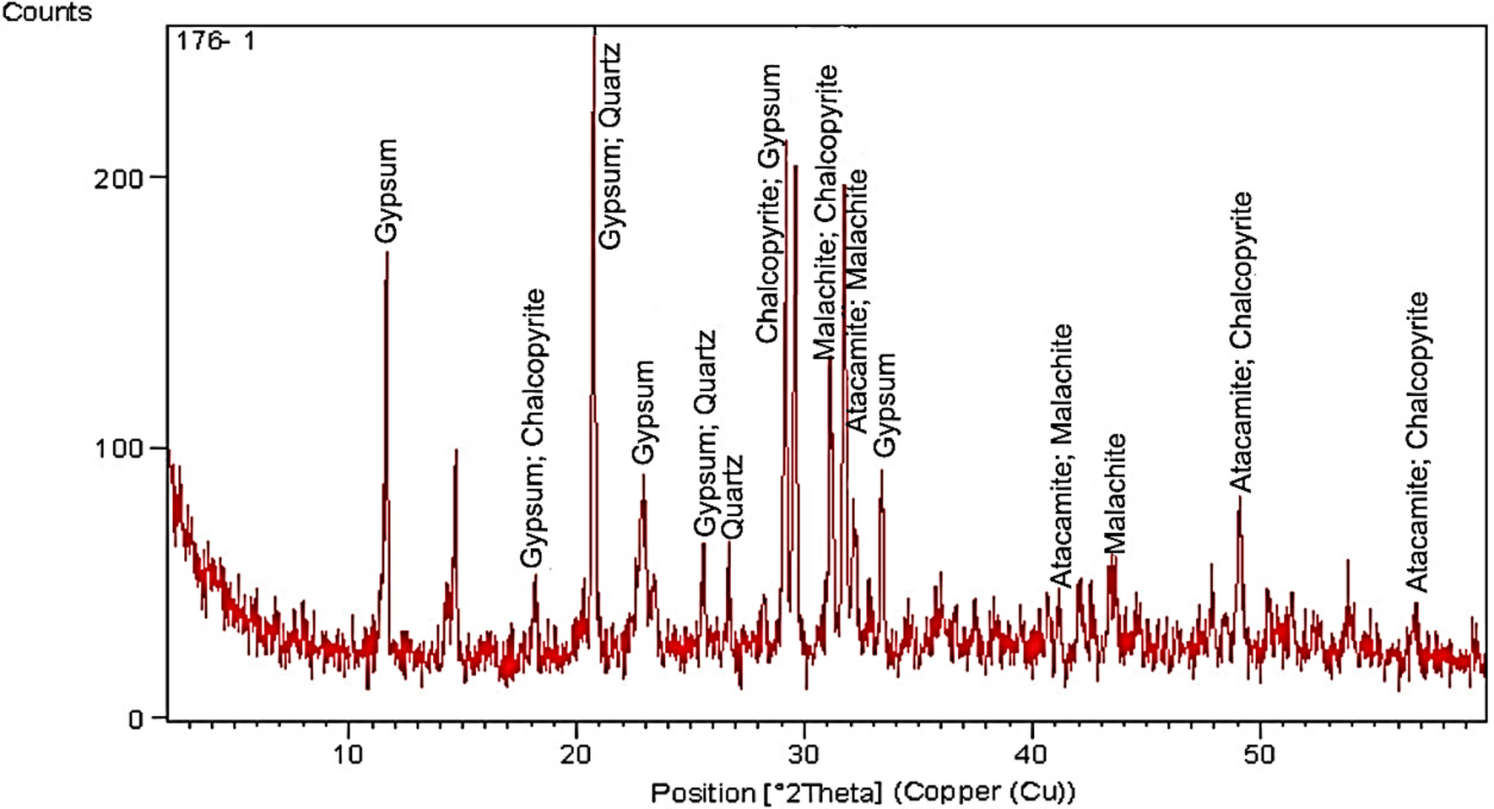
XRD spectrum of the corrosion products and dust deposits on the crescent’s surface.

X-ray diffraction of corrosion products and dust accumulated on the crescent revealed the presence of various corrosion compounds, such as malachite, atacamite, and chalcopyrite, and compounds resulting from dust and dirt, such as gypsum and quartz, as shown in Fig. 7, and Table 2. The brass crescent is exposed to varying humidity, temperature fluctuations, and contaminants, all of which contribute to the formation of these compounds, leading to accelerated deterioration and possible damage to the metal over time.

**Table 2:** Corrosion products and dust compounds were identified through XRD analysis.

Sample	Crystalline component	Compound name	Chemical formula	Ref. Code	Semi-quantitative %
Corrosion Products with accumulated dust	Malachite	Basic Copper Carbonate	Cu₂CO₃(OH)₂	00-056-0001	27
	Atacamite	Copper Hydroxide Chloride	Cu_2_(OH)_3_Cl	00-002-0146	11
	Chalcopyrite	Copper Iron Sulfide	CuFeS₂	01-075-0253	11
	Quartz	Silicon Oxide	SiO_2_	01-085-0795	12
	Gypsum	Calcium Sulfate Hydrate	CaSO_4_.2H_2_O	00-021-0816	39

Malachite (Cu_2_CO_3_(OH)_2_), a basic copper carbonate, forms when copper in the brass alloy reacts with carbon dioxide (CO_2_) in the atmosphere and moisture. The reaction typically occurs in outdoor environments where CO_2_ levels are elevated, leading to the formation of malachite as a green corrosion product. Atacamite (Cu_2_Cl(OH)_3_) is a basic copper chloride that forms in the presence of chloride ions, which can originate from salt in the air or from pollution. Chloride ions penetrate the brass surface, reacting with the copper and moisture to form atacamite. This compound is often associated with aggressive, localized corrosion and pitting. Chalcopyrite (CuFeS_2_), a copper iron sulphide, can form in the presence of sulphur compounds, which are common in industrial environments due to pollution from combustion processes. Sulfur reacts with copper and iron present in the alloy, leading to the formation of chalcopyrite. This process is often accelerated by the presence of moisture and acidic conditions. Gypsum (CaSO_4_·2H_2_O) and quartz (SiO_2_) are not direct corrosion products of Brass but result from the accumulation and calcification of dust and dirt on the surface of the crescent. Gypsum forms from the reaction of calcium in the dust with sulphur dioxide (SO_2_) in the atmosphere, especially in polluted environments. Quartz, being a common mineral in dust, can become embedded in the corrosion layers over time^45,46^.

Bird excrements, beyond their impact on the aesthetic appearance of brass objects, can cause chemical damage. Due to their acidic nature—primarily from uric acid—these droppings react with copper in Brass, forming corrosive salts that promote corrosion (Fig. 3). Additionally, bird excreta retain moisture, facilitating electrochemical reactions that accelerate corrosion. Additionally, the organic material in the droppings breaks down over time, producing more acids that further attack the copper surface. This can result in localized corrosion, patina formation, and even crevice corrosion if the droppings are left on the metal for extended periods^47–49^.

### Chemical composition of PKO

**Fig. 8:**
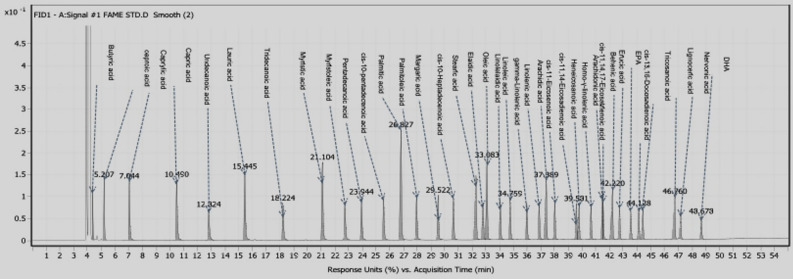
Gas chromatography analysis of palm kernel oil illustrates the distribution and retention times of key fatty acid methyl esters, with lauric acid being the predominant component.

The chromatogram from the GC analysis identified various fatty acid methyl esters (FAMEs) in the palm kernel oil sample. The key components and their retention times (RT), peak areas, and relative percentages are summarized in Table 3 and Fig. 8. Fatty acids, such as palmitic (16:0), stearic (18:0), and oleic (18:1), are common in animal and vegetable fats and oils. Unlike palm oil, which contains balanced amounts of saturated and unsaturated fats, palm kernel oil is primarily saturated. It is rich in lauric acid, myristic acid, palmitic acid, and oleic acid^50^.

**Table 3:** Gas chromatography results of palm kernel oil.

Peak	Retention time (RT)	Component	Area %
1	4.32	FAME-1	0.25
2	5.12	FAME-2	4.13
3	10.22	FAME-3	10.42
4	24.05	FAME-4	52.63
37	48.95	FAME-37	16.46

The gas chromatography analysis revealed that the palm kernel oil primarily consists of fatty acid methyl esters (FAMEs), with several peaks indicating the presence of saturated and unsaturated fatty acids. The highest concentration components include Peak 4, representing 52.63% of the total area, which is likely a major saturated fatty acid such as lauric acid commonly found in palm kernel oil. The composition aligns with the typical profile of commercial palm kernel oil, which is rich in medium-chain fatty acids (such as lauric and myristic acids) known for their stability and beneficial properties in various industrial applications. Given the oil’s chemical composition, the commercially sourced oil appears to be of good quality and suitable for use as a corrosion inhibitor as tested in electrochemical experiments. Commercial palm kernel oil already meets the necessary standards for performance, containing the essential fatty acid profile.

### Corrosion tests

**Fig. 9:**
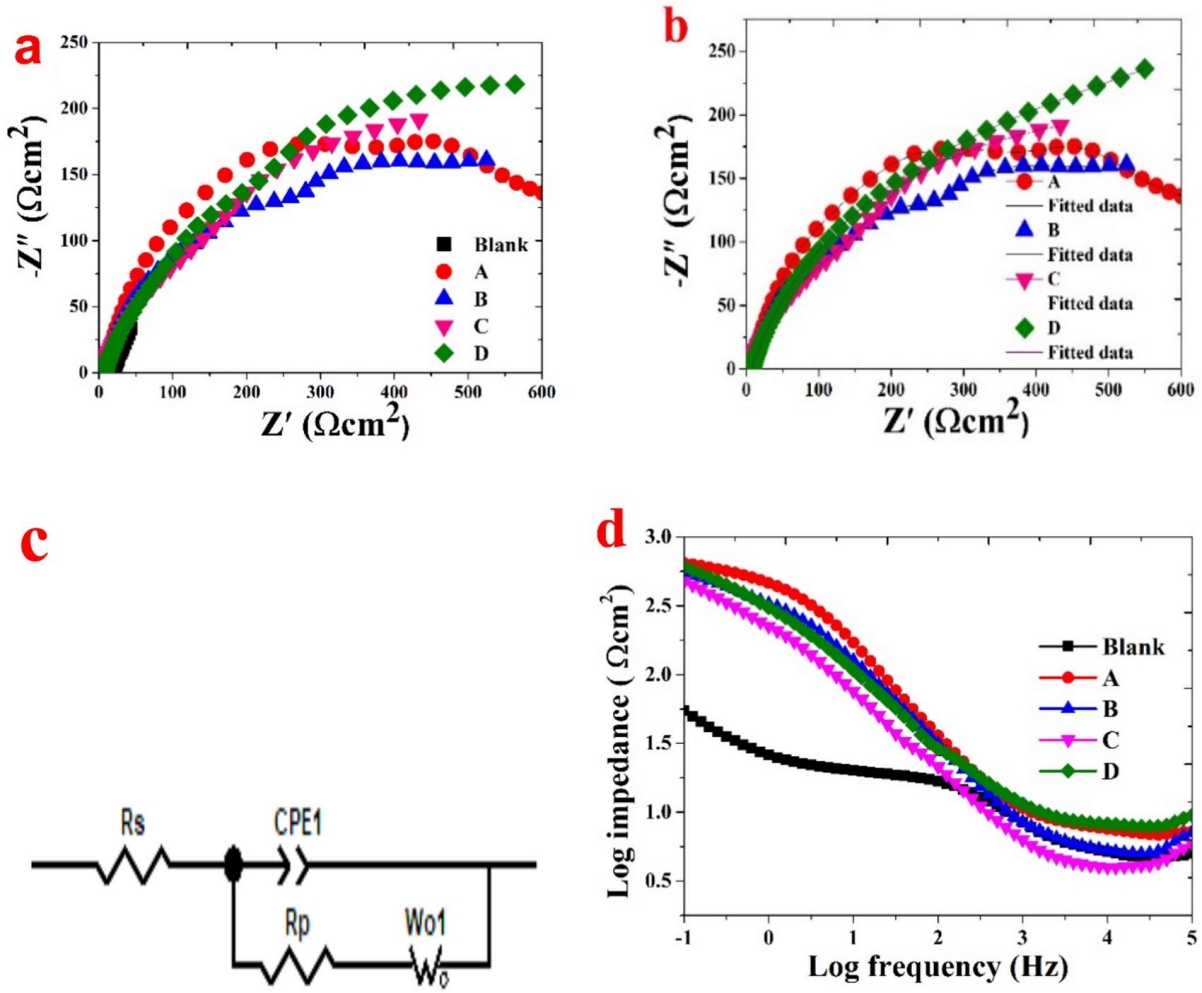
(**a**) Nyquist plots, (**b**) EIS fitting plots for PKO samples with varying concentrations, (**c**) the equivalent electrical circuit model, (**d**) Bode impedance plots understanding the corrosion behavior of Brass in a 3.5% sodium chloride solution both with and without various concentrations of palm kernel oil as a corrosion inhibitor.

The study evaluated how corrosion attack was affected by adding and increasing the concentration of PKO inhibitor to a brass alloy^51^. The corrosion behavior of the samples in a NaCl-based solution, as determined by EIS and the corresponding electrical circuit model, is shown in Fig. 9a–c. The data reveal that D (7%) exhibits the highest corrosion resistance, followed by C (5%), B (3%), and A (1%) as the least effective. Table 4 presents the reference parameters derived from EIS fitting plots, as shown in Fig. 9b, using manual adjustments in Zview software for PKO samples with varying concentrations. The results confirm that all PKO samples (A–D) demonstrate superior corrosion resistance compared to the blank. The dense and stable surface of the PKO samples likely contributes to their high corrosion resistance, reflected by the larger capacitive response (Fig. 9). In contrast, the blank sample experienced significant deterioration due to the formation of a corrosion product layer on its surface, further highlighting the effectiveness of PKO in corrosion inhibition.

**Table 4:** The reference parameters were extracted from the EIS fitting plots for PKO samples with varying concentrations.

Sample	R_s_ (Ω) Error%	R_p_ (Ω) Error%	CPE1-T Error%	CPE1-P Error%	Wo1-R Error%	Wo1- T Error%	Wo1-P Error%	Chi-squared
A 1%PKO	7.104	620	0.00020529	0.7701812	2710	4.004	0.56636	0.0024493
	1.2%	1.9%	3.4%	0.7%	2.17%	3.71%	3.2%	
B 3%PKO	5.32	503.7	0.00039787	0.7036911	1913	4.333	0.54813	0.011338
	2.8%	4.5%	7.5%	1.7%	2.3%	4.2%	3.2%	
C 5%PKO	4.186	498.6	0.00069834	0.69008	1343	4.788	0.55316	0.01853
	3.9%	7.8%	1.0%	2.5%	2.0%	3.9%	2.7%	
D 7%PKO	7.69	509.1	0.00060333	0.64772	1810	5.802	0.51893	0.004334
	1.7%	4.5%	4.4%	1.1%	1.9%	4.0%	2.1%	

The bigger capacitive response suggests that the D sample (highest concentration of PKO 7%) dense and stable surface may offer significant protection against corrosive environments. The Nyquist plots align with the bode impedance measurements, showing consistent behavior, as shown in Fig. 9c. The efficacy of palm kernel oil (PKO) as a corrosion inhibitor for Brass in a 3.5% NaCl solution at room temperature is clearly trended by the electrochemical data in Tables 4 and 5. In Electrochemical Impedance Spectroscopy (EIS), the fitting results include key parameters such as R_s_ (solution resistance) and R_p_ (polarization resistance), which are inversely related to I_corr._. CPE-T and CPE-P are parameters of the Constant Phase Element (CPE) that provide detailed insights into the electrochemical behavior, as summarized in Table 4. The chi-squared value evaluates the fit quality between experimental and modelled data, with lower values indicating a better fit and closer alignment between measured and simulated impedance values. The sum of squared residuals, derived from differences between observed and predicted values, typically varies, but values nearing zero signify excellent model performance, as demonstrated in Table 4 for all PKO samples, which reveal notable electrochemical behavior. Wo1-T, Wo1-R, and Wo1-P are specific parameters of a finite-length Warburg element. Wo1-R represents the resistance associated with the solid electrolyte interface and charge-transfer processes. Wo1-T corresponds to the diffusion layer thickness, influencing ion movement through the electrolyte. Wo1-P, an exponent modifying the phase angle, reflects deviations from ideal diffusion behavior due to changes in the electrolyte. These parameters offer a comprehensive understanding of the system’s electrochemical dynamics.

**Table 5:** Electrochemical parameters for blank and varying concentration samples.

Sample	R_p_ (Kohm cm^2^)	Beta a (mV)	Beta c (mV)	E(i = 0) (mV)	Corrosion rate (C.R.) (µm/y)	I_corr_ _(µA/cm_^2^_)_
Blank	0.047	201.3	− 183.2	25.7	7559.5	653
A 1%PKO	1.62	50.9	− 297.3	− 200.0	165.13	14.3
B 3%PKO	2.62	79.2	− 162.5	− 242.4	78.668	6.7954
C 5%PKO	2.59	58.3	− 98.4	− 247.8	54.566	4.7134
D 7%PKO	3.27	40.2	− 84.8	− 242.5	20.696	1.7878

As a result, by raising the concentration, inhibitor addition lowers the electrochemical activity on the metal surface up to 99.7% corrosion inhibition at optimal levels^51^. Additionally, these protective layers by (PKO) reduce the dissolution of brass ions in aggressive environments, significantly slowing corrosion rates^52^. Palm kernel oil (PKO) prevents corrosion in copper alloys principally by adsorbing onto the metal surface, thereby slowing electrochemical processes and reducing the likelihood of pitting, cracking, and void formation. This mechanism involves PKO molecules forming a protective barrier that restricts the passage of corrosive ions to the metal surface while also preventing metal ions from dissolving into the solution^51^. The effectiveness of PKO as a corrosion inhibitor lies in its ability to alter the electrochemical properties at the metal-solution interface, leading to significantly reduced corrosion rates^52^.

Samples with and without varying concentrations of PKO were analyzed to investigate the corrosion mechanism. Corrosion parameters, including current density (I_corr._), anodic Tafel slope (βa), cathodic Tafel slope (− βc), and corrosion potential (E_corr._), were determined using the Tafel extrapolation method.

**Fig. 10:**
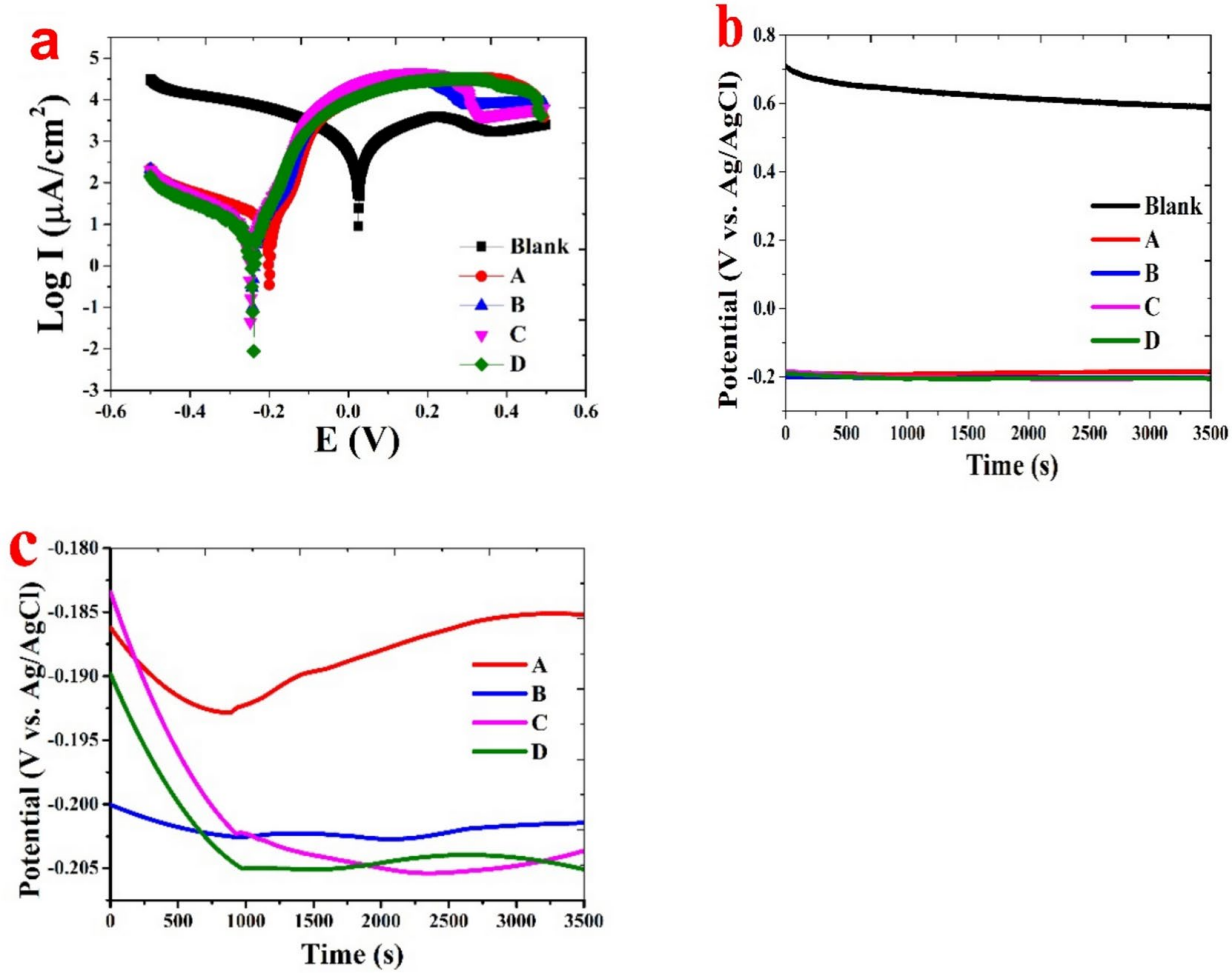
(**a**) Potentiodynamic polarization curves and (**b**,**c**) OCP curves, illustrating the effect of various concentrations of palm kernel oil (PKO) as corrosion inhibitor on the corrosion resistance of brass alloy in a 3.5% NaCl solution.

The corrosion rate was ascertained using the Tafel extrapolation measurements, as seen in Fig. 10a. The following formula is used to determine the C.R.

1 $$\text{C}.\text{R}. = \frac{0.0032 \times \text{I}_\text{corr }\times (\text{M}.\text{W}.)}{\text{n }\times \text{ d}}$$

where M.W. refers to the molecular weight of the corroded material (g/mol), while C.R. denotes the corrosion rate (mpy). Additionally, Icorr represents the current density of corrosion (A cm^−2^), n indicates the number of charge transfers throughout the corrosion process, and d corresponds to the density (g cm^−3^)^52^.

According to the data, the corrosion current density (I_corr._) dropped during the testing period, and the D sample had the lowest corrosion rate (C.R.). As the PKO concentration rose, when compared to a blank sample, the Tafel plot showed a drop in E_corr._, I_corr._, and C.R., as shown in Table 5 and Fig. 10a. The study demonstrates that, in comparison to a blank sample, increasing the PKO content results in a drop in I_corr._. On the other hand, PKO increases corrosion resistance in brass alloys. Based on the electrochemical data, the polarization resistance (R_p_) values progressively increase with higher concentrations of palm kernel oil (PKO), reflecting improved protection against corrosion^53^. The blank sample exhibits the lowest R_p_ value at 0.047 kΩ cm^2^, while the 7% PKO sample reaches the highest R_p_ at 3.27 kΩ cm^2^, indicating a significant enhancement in resistance to corrosion as the inhibitor concentration rises. A similar pattern is seen in the corrosion current density (I_corr._) (µA/cm^2^), where the blank sample demonstrates a very high I_corr._ value of 653 µA/cm^2^, signaling severe corrosion. However, with the introduction of PKO, the current density significantly decreases, reaching its lowest value at 1.7878 µA/ cm^2^ with 7% PKO, which clearly indicates that the inhibitor reduces the electrochemical activity on the metal surface, thereby slowing down the corrosion process^54,55^.

**Fig. 11:**
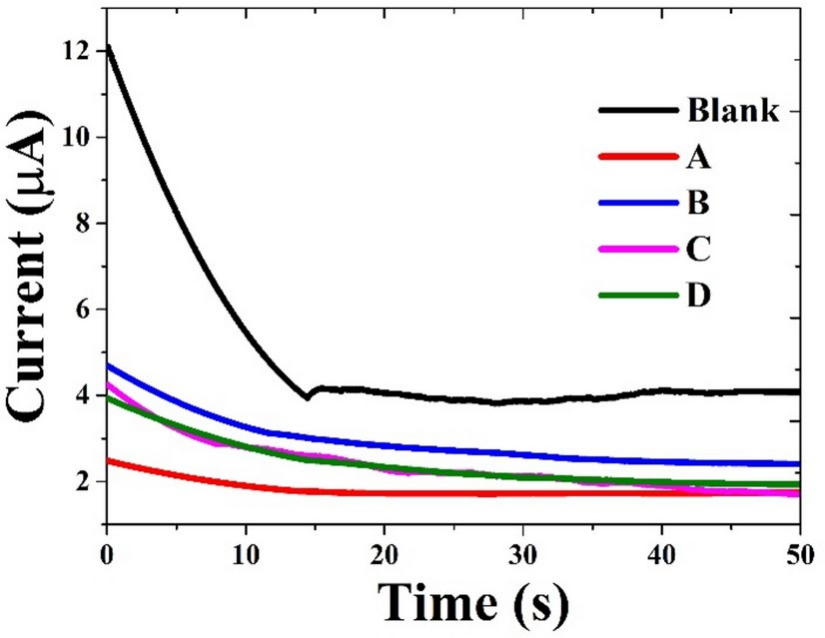
Current transient curves of Brass in a 3.5% sodium chloride solution with and without various concentrations of palm kernel oil as a corrosion inhibitor.

Likewise, the corrosion rate (µm/y) follows a similar trend to that of I_corr._. The blank sample shows the highest corrosion rate at 7559.5 µm/year, but as the concentration of PKO increases, the corrosion rate decreases, reaching 20.696 µm/year at the 7% PKO concentration, demonstrating the effectiveness of PKO in mitigating corrosion^55^. Regarding the anodic and cathodic slopes (Beta a, Beta c), the anodic slope (Beta a) generally decreases with the increase in PKO concentrations, which suggests that the inhibitor promotes the formation of a more protective passive layer on the metal surface, thereby reducing the anodic dissolution^56^. Similarly, the cathodic slope (Beta c) shows variations, indicating that the inhibitor suppresses cathodic reactions, likely due to its effect on oxygen reduction or hydrogen evolution. The results validate the effectiveness of the PKO inhibitor in NaCl solution, as evidenced by the shift of E_corr._ toward the y-axis, indicating a significant role of the inhibitor in the cathodic reaction mechanism of the copper alloy, thereby reducing metal dissolution^56^, as shown in Fig. 10a. In line with other electrochemical measurements, as shown in Figs. 9, 10 and 11 the polarization data reveal that the D sample exhibits the highest corrosion inhibition performance, demonstrating its ability to lower the C.R. of the copper alloy surface through adsorption and/or the formation of a protective film on active sites^51^.

The open circuit potential (OCP) curves for the blank sample and different palm kernel oil (PKO) concentrations in a 3.5% NaCl solution are displayed in Fig. 10b. This indicates how PKO affects the samples’ electrochemical performance and corrosion behavior. As PKO concentration rises, OCP values shift toward greater negative potentials, which confirms Tafel parameters and plots. By slowing down anodic and cathodic reactions, inhibitors (PKO) improve the metal surface’s protective layer and prevent corrosion at its active sites^51^. As a result of decreased electron flow associated with corrosion processes, the potential becomes increasingly negative as C.R. decreases^54,55^. In this regard, using an inhibitor effectively can greatly increase the resistance to corrosion in brass alloys.

## Conclusions

The study of the brass crescent has provided valuable insights into its material composition, historical manufacturing techniques, and ongoing conservation challenges. The analysis confirmed that the crescent is a yellow brass alloy (64.77% copper, 33.65% zinc, 1.58% tin), classified as alpha Brass, known for its ductility and corrosion resistance. However, corrosion products such as malachite, atacamite, and chalcopyrite reveal the impact of environmental factors on its degradation, primarily due to electrochemical reactions with pollutants, moisture, and dust. Selective corrosion processes like dezincification were observed, leading to a porous copper-rich structure that compromises the alloy’s integrity. SEM analysis also identified zinc white globules and stress corrosion cracking (SCC), particularly in polluted environments, emphasizing the vulnerability of high-zinc Brass to SCC. Incorporating electrochemical tests, palm kernel oil has been demonstrated to be an effective corrosion inhibitor, with efficiency improving at higher concentrations, as shown by enhanced polarization resistance, lower corrosion current density, and reduced corrosion rates by 99.7%. The gas chromatography analysis of the palm kernel oil confirmed it is rich in fatty acid methyl esters, with lauric acid as the predominant component, aligning with the chemical profile of high-quality commercial oil. This suggests that commercial palm kernel oil is suitable for corrosion inhibition, and laboratory extraction is unnecessary for the current application. These findings highlight the need for targeted conservation strategies to mitigate further corrosion and ensure the long-term stability of the crescent, given its historical and cultural significance.

## Data Availability

Data availability The datasets used and/or analysed during the current study available from the corresponding author on reasonable request.
